# MicroRNA Profiling in Human Colon Cancer Cells during 5-Fluorouracil-Induced Autophagy

**DOI:** 10.1371/journal.pone.0114779

**Published:** 2014-12-19

**Authors:** Ni Hou, Jia Han, Jie Li, Yingxun Liu, Yannan Qin, Lei Ni, Tusheng Song, Chen Huang

**Affiliations:** 1 Department of Genetics and Molecular Biology, Xi'an Jiaotong University School of Medicine, Xi'an, China; 2 Department of General Surgery, the Second Affiliated Hospital, Xi'an Jiaotong University School of Medicine, Xi'an, China; 3 Key Laboratory of Environment and Genes Related to Diseases, Xi'an Jiaotong University School of Medicine, Xi'an, China; 4 Cardiovascular Research Center, Xi'an Jiaotong University School of Medicine, Xi'an, China; Roswell Park Cancer Institute, United States of America

## Abstract

Autophagy modulation is now recognized as a potential therapeutic approach for cancer (including colorectal cancer), yet the molecular mechanisms regulating autophagy in response to cellular stress are still not well understood. MicroRNAs (miRNAs) have been found to play important roles in controlling many cellular functions, including growth, metabolism and stress response. The physiological importance of the miRNA-autophagy interconnection is only beginning to be elucidated. MiRNA microarray technology facilitates analysis of global miRNA expression in certain situations. In this study, we explored the expression profile of miRNAs during the response of human colon cancer cells (HT29s) to 5-FU treatment and nutrient starvation using miRNA microarray analysis. The alteration of miRNA expression showed the same pattern under both conditions was further testified by qRT-PCR in three human colon cancer cell lines. In addition, bioinformatic prediction of target genes, pathway analysis and gene network analysis were performed to better understand the roles of these miRNAs in the regulation of autophagy. We identified and selected four downregulated miRNAs including hsa-miR-302a-3p and 27 upregulated miRNAs under these two conditions as having the potential to target genes involved in the regulation of autophagy in human colon cancer cells. They have the potential to modulate autophagy in 5-FU-based chemotherapy in colorectal cancer.

## Introduction

5-fluorouracil (5-FU)-based adjuvant chemotherapy has been widely used as the mainstream for the treatment of colorectal cancer (CRC). However, because of the resistance to 5-FU in many patients, novel therapeutic strategies are being explored [1]. Autophagy is an evolutionarily conserved eukaryotic process that maintains intracellular homeostasis by eliminating unnecessary proteins and damaged or aged organelles [2]. In the past decades, accumulating evidence has shown that autophagy is extensively associated with cancer [3]. By maintaining cellular homeostasis in healthy cells, autophagy prevents tumoral transformation. Autophagy is also important for tumor progression, allowing tumor cells to survive metabolic stress or anoikis, sustaining their adaptation to reprogrammed metabolism, supporting tumor development by inducing dormancy and maintaining the survival and self-renewal of cancer stem cells. Moreover, because autophagy plays essential roles in determining how tumor cells respond to therapy, autophagy modulation is recognized as a potential therapeutic approach in cancer [4], [5]. Autophagy seems to represent a valid mechanism of resistance against radio- and chemotherapy. Our previous studies showed that inhibition of autophagy by 3-methyladenine (3-MA) or small interference RNA targeting Atg7 (Atg7 siRNA) augmented the efficiency of 5-FU by enhancing apoptosis in human colon cancer [6], [7]. Autophagy is highly conserved and tightly regulated. However, the molecular mechanisms regulating autophagy in response to cellular stress are still not well understood.

MicroRNAs (miRNAs), 18–25 nucleotides in length, are endogenous small, noncoding RNAs that regulate the expression of their target genes by inhibiting translation or cleaving messenger RNA (mRNA), mainly through interaction at the 3' untranslated regions (UTRs) of the target mRNAs [8]. MiRNAs can simultaneously regulate a multitude of targets and biological networks. Conversely, several different miRNAs can bind to and cooperatively control a single mRNA target. MiRNAs have been found to play important roles in controlling many cellular functions, including growth, differentiation, metabolism and stress response and provided a clear advantage from a clinical viewpoint [9]–[11]. In recent years, some miRNAs have been studied as mediators of autophagy regulation. MiRNA-30a can sensitize hepatoma cells to cisplatin by targeting beclin-1-mediated autophagy [12]. MiRNA-101 has been demonstrated to be as a potent inhibitor of autophagy induced by etoposide or rapamycin in breast cancer cells [13]. Jegga et al. also proposed that miRNA-130, miRNA-98, miRNA-124, miRNA-204 and miRNA-142 have potential regulatory functions in the autophagic process based on computational analysis [14]. The physiological importance of the miRNA-autophagy interconnection is only beginning to be elucidated.

Because of the large number of miRNAs, miRNA microarray technology has been extensively applied to determine global miRNA expression in certain situations [15]. In this study, we explored the expression profile of miRNAs in the response of human colon cancer cells (HT29s) to 5-FU treatment using miRNA microarray analysis. To prioritize the miRNAs that correlated with autophagy, autophagy was also induced by a second means (nutrient starvation), and the miRNA expression was also observed in that context. The altered miRNA expression showed a same pattern under both conditions was further testified by qRT-PCR in three human colon cancer cell lines. In addition, bioinformatics prediction of target genes, pathway analysis and gene ontology network analysis were also performed to better understand the roles of these miRNAs in the regulation of autophagy. We identified and selected four downregulated miRNAs and 27 upregulated miRNAs upon 5-FU treatment and starvation in human colon cancer cells. These 31 miRNAs have the predicted target genes of the regulation of autophagy, including autophagy core genes and autophagy regulators and have the potential to modulate autophagy in 5-FU-based chemotherapy in CRC.

## Materials and Methods

### Materials

5-FU was purchased from Sigma (Sigma-aldrich, Saint Louis, MO). LC3 polyclonal antibody was purchased from MBL (MBL, Nagoya, Japan). Anti-p62 and anti-β-actin antibodies were obtained from Sigma, and the mTOR antibody was from Cell Signaling Technology (Cell signaling technology, Danvers, MA).

### Cell culture and treatment

HT29, HCT116 and DLD1 human colorectal carcinoma cells were purchased from the American Type Culture Collection, kindly provided by Prof. Kuwano and cultured in RPMI-1640 medium supplemented with 10% fetal bovine serum at 37°C in a humidified atmosphere of 5% CO_2_/95% air with medium changes every two days. Cells in mid-log phase were used in this study. For 5-FU treatment, HT29s were treated with 5 µM of 5-FU for 24 h. For nutrient starvation, HT29s were incubated in Krebs-Ringer buffer [16] (120 mM NaCl, 5 mM KCl, 24 mM NaHCO_3_, 5.6 mM glucose, 2 mM CaCl_2_, pH 7.6) at 37°C for 7 h.

### Measurement of cell viability and apoptosis

Cell viability was determined using Cell Counting Kit 8 (CCK-8). Cells were seeded in 96-well flat bottom microtiter plates at a density of 1×10^3^ cells per well. After treatment, 10 µl of the CCK-8 solution was added to each well and incubated at 37°C for 1 h. The absorbance of the solution was read spectrophotometrically at 450 nm with a reference at 650 nm using a microtiter plate reader (BIO-TEK ELX800). Cell viability was calculated according to the following formula: cell viability (%)  = A450 (sample)/A450 (control) ×100.

Cell apoptosis was assayed using the Apoptosis Detection kit. Briefly, cells were harvested and stained with Annexin V –FITC (Annexin V) and propidium iodide (PI). Apoptosis was defined by Annexin V^+^/PI^-^ (early apoptosis) and Annexin V^+^/PI^+^ (late apoptosis) as determined by FACScan (Becton Dickinson).

### Analysis of autophagy

Analysis of autophagy was performed mainly by immunofluorescence and immunoblotting for microtubule-associated protein 1B-light chain 3 (LC3) as described previously [7], [16]. To determine the immunofluorescence of LC3, cells on the chamber slide were fixed with 4% paraformaldehyde and permeabilized with 0.05% Triton X-100. After blocking, cells were incubated with an anti-LC3 antibody (1∶500 dilution) at 4°C overnight and, incubated with Alexa Fluor 488-conjugated anti-rabbit antibody after washing. Slides were mounted and examined using a fluorescence microscope (ECLIPSE TE2000-U, Nikon). Staining with 0.1 µg/ml 4′, 6-diamidino-2-phenylindole (DAPI) was performed for identifying nucleus. For immunoblotting of LC3, 20 µg of cell lysate was separated on a 5–20% Tris-Tricine Ready Gel SDS-PAGE (Bio-Rad) for polyvinylidene difluoride (PVDF) membrane blotting. The blotted membrane was blocked and incubated with anti-LC3 (1∶1000 dilution). The immunoreactive bands were visualized by advanced chemiluminescence using horseradish peroxidase-conjugated anti-rabbit antibody (1∶5000 dilution). p62 and mTOR immunoblotting (1∶1000 dilutions both) were also performed to evaluate the autophagy state.

### RNA isolation and miRNA microarray

Total cellular RNA was harvested using TRIzol (Invitrogen, Carlsbad, CA) and a miRNeasy mini kit (Qiagen, GmbH, Hilden, Germany) according to the manufacturer's instructions. Exiqon LNA MicroRNA Human Array including all human mature miRNAs (Database 18.0) was used to profile miRNA expression and performed by KangCheng Bio-Tech Inc. (Shanghai, China). We did the submission of our microarray data to Gene Expression Omnibus, and the accession number is GSE61943. In brief, RNA samples (1 µg) were labeled using a miRCURY Hy3 labeling kit and hybridized on the miRCURY LNA Array (v.18.0). Following washing, the slides were scanned using an Axon GenePix 4000B microarray scanner, and the raw intensity of the image was read and analyzed using GenePix pro 6.0 software (Axon). Four replicated spots of each probe on the same slide were averaged. Expressed miRNA data were normalized using the Median normalization. After normalization, differentially expressed miRNAs were identified through Fold Change filtering (> = 2).

### Real-time qRT-PCR analysis for miRNA expression

Quantitative reverse transcription polymerase chain reaction (qRT-PCR) was performed to validate the miRNA array data. Mature miRNAs were reverse transcribed into cDNA by stem-loop reverse transcription using the PrimeScript RT reagent kit (Takara Bio, Shiga, Japan) and specific stem-loop primers as shown in Table 1. qRT-PCR for each miRNAs was performed using FastStart Essential DNA Green Master (Roche Molecular Biochemicals, Mannheim, Germany) on an lightcycle96 machine (Roche) according to the manufacturer's instructions. The primers used are listed in Table 1. Each sample was analyzed in triplicate. The miRNA expression levels were normalized to and quantified by U6 RNA. Relative quantitation was calculated using the 2^(−**ΔΔ**Ct)^ method.

**Table 1:** Primers used in this work.

Name	Sequence (5′ → 3′)
hsa-miR-302a-3p RT	GTCGTATCCAGTGCGTGTCGTGGAGTCGGCAATTGCACTGGATACGACTCACCAAAAC
hsa-miR-302a-3p-F	ATCCAGTGCGTGTCGTGGA
hsa-miR-302a-3p-R	CGACGTAAGTGCTTCCATG
hsa-miR-548ah-5p RT	GTCGTATCCAGTGCGTGTCGTGGAGTCGGCAATTGCACTGGATACGACCAAACAC
hsa-miR-548ah-5p-F	ATCCAGTGCGTGTCGTGGA
hsa-miR-548ah-5p-R	CTCGTGCAAAAGTGATTGCA
hsa-miR-30a-5p RT	GTCGTATCCAGTGCGTGTCGTGGAGTCGGCAATTGCACTGGATACGACCTTCCAGTCG
hsa-miR-30a-5p-F	ATCCAGTGCGTGTCGTGGA
hsa-miR-30a-5p-R	AGTCGACTGTAAACATCCTC
hsa-miR-23a-3p RT	GTCGTATCCAGTGCGTGTCGTGGAGTCGGCAATTGCACTGGATACGACGGAAATCCCT
hsa-miR-23a-3p-F	ATCCAGTGCGTGTCGTGGA
hsa-miR-23a-3p-R	GTCACCGATCACATTGCCA
hsa-miR-195-5p RT	GTCGTATCCAGTGCGTGTCGTGGAGTCGGCAATTGCACTGGATACGACGCCAATATT
hsa-miR-195-5p-F	ATCCAGTGCGTGTCGTGGA
hsa-miR-195-5p-R	CTAGCTCTAGCAGCACAGA
hsa-Let-7c-5p RT	GTCGTATCCAGTGCGTGTCGTGGAGTCGGCAATTGCACTGGATACGACAACCATACA
hsa-Let-7c-5p-F	ATCCAGTGCGTGTCGTGGA
hsa-Let-7c-5p-R	CGACGTTGAGGTAGTAGGTT
U6 RT	CGCTTCACGAATTTGCGTGTCAT
U6-F	GCTTCGGCAGCACATATACTAAAAT
U6-R	CGCTTCACGAATTTGCGTGTCAT

### Target prediction and function analysis

The target genes of the miRNAs were predicted using the intersection of two major online miRNA target prediction algorithms, TargetScan (http://www.targetscan.org) [17] and PicTar (http://pictar.mdc-berlin.de) [18], or TargetScan and miRDB (http://mirdb.org) [19] if there was no data for some miRNAs in PicTar.

DIANA-miRPath v2.0 (http://www.microrna.gr/miRPathv2) was applied to analyze the main functions of miRNAs [20]. Generally, miRNA and pathway-related information was obtained from miRBase and the Kyoto Encyclopedia of Genes and Genomes (KEGG) v58.1, respectively. A one-tailed Fisher's exact test was used to identify the enriched KEGG pathways with targets of specific miRNAs, and the false discovery rate (FDR) was calculated to correct the p value. Enrichment provides a measure of the significance of the function; as the enrichment increases, the corresponding function is more significant.

Gene Ontology (GO) network analysis was also used to analyze the main function of the predicted target genes and uncover the miRNA-gene regulatory network on the basis of biological processes and molecular functions. The CyTargetLinker plugin in Cytoscape (http://projects.bigcat.unimaas.nl/cytargetlinker) was used to construct an integrative network of the miRNA–target interactions for the six miRNAs identified in our study [21], [22]. The validated targets for each miRNA were obtained from mirTarBase, and the predicted targets were obtained from Targetscan.

### Statistical analysis

All data were expressed as means ± standard deviation (SD). All statistical analyses were performed using SPSS version 17.0 software. p<0.05 was considered to be statistically significant.

## Results

### 5-FU decreased the viability of HT29 human colon cancer cells,

**Figure 1:**
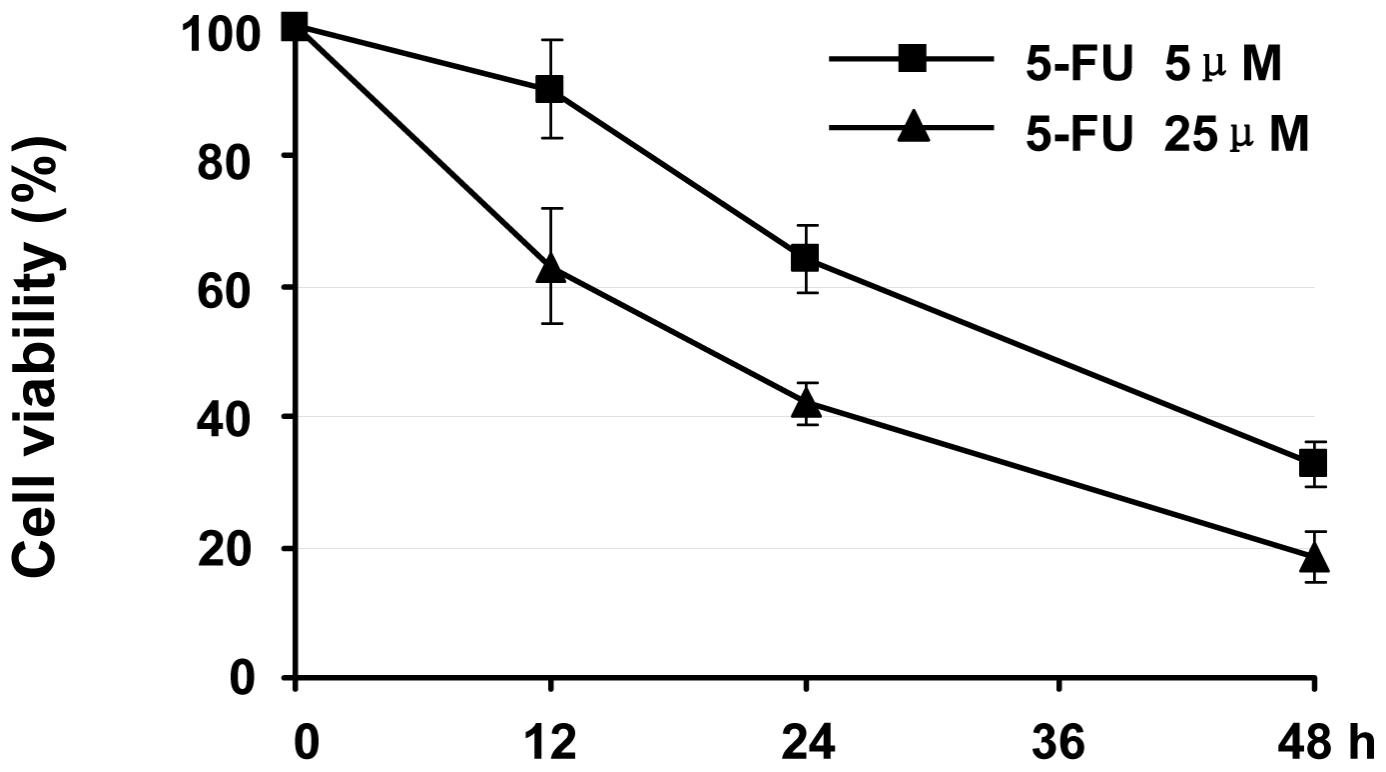
Effect of 5-FU on the viability of HT29 human colon cancer cells. HT29 cells were incubated with different concentrations (5 µM and 25 µM) of 5-FU for 12, 24, and 48 h. Cell viability was measured by CCK-8 assay. Data are shown as the mean ±SD.

The effect of the main CRC chemotherapy, 5-FU, in HT29 human colon cancer cells was confirmed by a CCK-8 assay. 5-FU (0∼500 µM) produced a dose- and time-dependent inhibition of cell viability that reached 64.20% and 42.20% after 5 µM 5-FU treatment for 24 h and 48 h, respectively (Fig. 1). 5-FU could have many effects on HT29 cells. Flow cytometry of Annexin V and PI staining was used to detect apoptosis in our experiment. There have little apoptosis in HT29 cells by 5 µM 5-FU treatment for 24 h (S1 Fig.).

### 5-FU induced the activation of autophagy in HT29 cells

**Figure 2:**
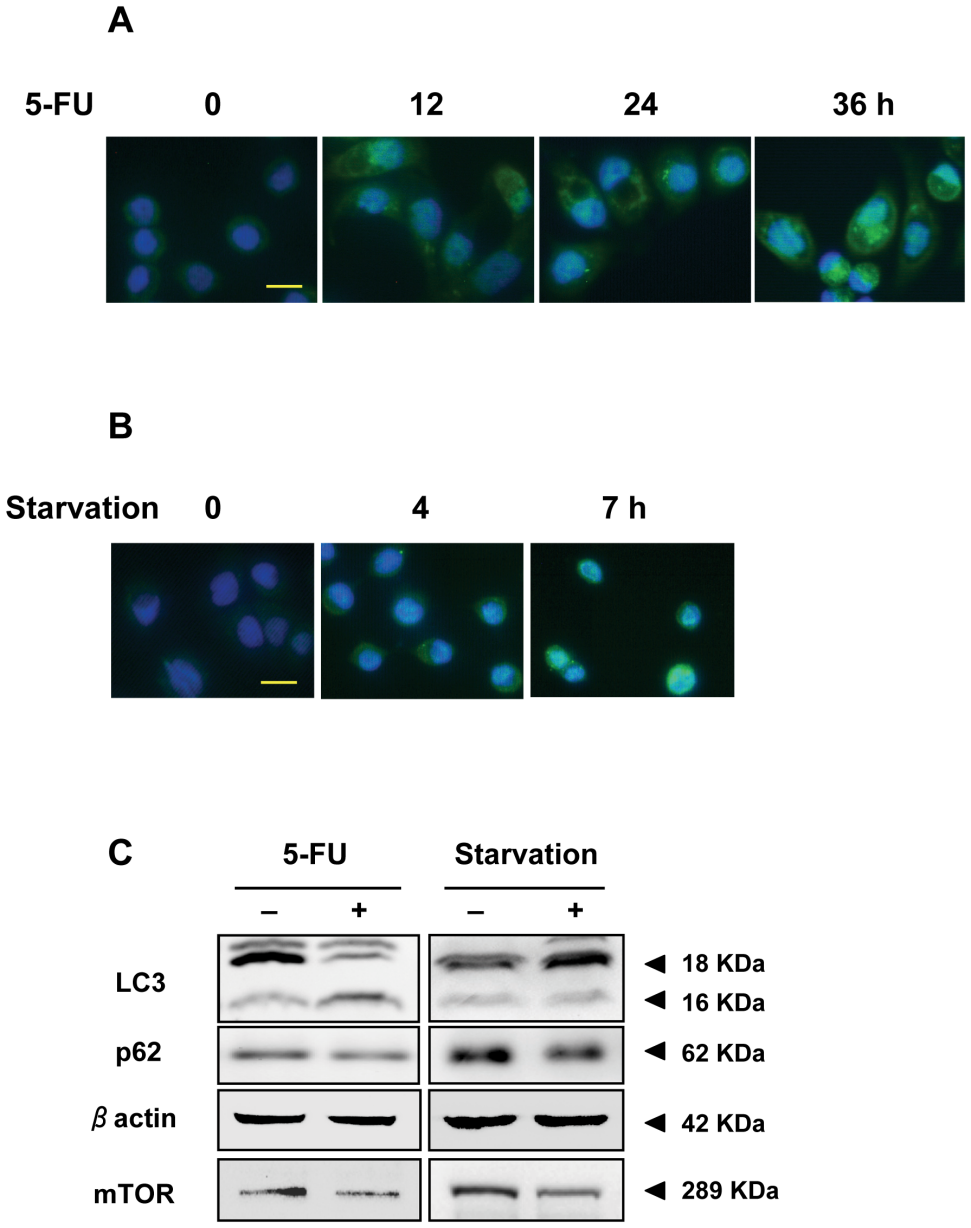
Autophagy is activated by 5-FU treatment and starvation in HT29 cells. HT29 cells were treated with 5 µM of 5-FU or not. Activation of autophagy was observed by LC3 immunofluorescence (A). HT29 cells were starved in Krebs-Ringer buffer or not. Activation of autophagy was observed by LC3 immunofluorescence (B). DAPI staining was performed for identifying nucleus. LC3, p62 and mTOR immunoblotting was performed using the lysates of HT29 cells treated by 5 µM of 5-FU for 24 h or not, and starved for 7 h or not (C). Data are the representative of three independent experiments. Bar, 20 µm.

Activation of autophagy by 5-FU in HT29 cells was detected by LC3 immunofluorescence. In the control cells, the distribution of LC3 showed a diffuse pattern (cytoplasm, LC3- I). 5-FU treatment altered the LC3 distribution to many coarse dots and punctate staining (Fig. 2A), and as time increased, the dots became more intense. The LC3-positive punctuates (LC3-II) represent autophagosomes. LC3 immunoblotting was also used to observe autophagy (Fig. 2C). LC3-II (16 kDa) was induced by 5 µM 5-FU treatment for 24 h. As a characteristic mechanism of inducing autophagy induction, nutrient starvation was also performed (Fig. 2B). Starvation made the LC3 staining more intense, and in 7 h, there was some punctuate staining. Additionally, the intensity of LC3 was increased by starvation for 7 h. As the indicator of autophagy flux, p62 was decreased both by 5-FU treatment and starvation (Fig. 2C). After 5 µM 5-FU treatment for 24 h, the cell viability of HT29 cells was inhibited, autophagy was activated, and there was almost no apoptosis. Then, we performed global expression profiling by miRNA microarray assays on both HT29 cells starved for 7 h and HT29 cells treated with 5 µM 5-FU for 24 h.

### Identification of altered miRNA expression by 5-FU and starvation in HT29 cells

After microarray scanning and normalization, 124 out of 1900 mature human miRNAs were identified as upregulated by starvation for 7 h in HT29 cells, and 56 miRNAs were downregulated. With 5 µM 5-FU treatment for 24 h, there were 302 upregulated miRNAs and 86 downregulated miRNAs in HT29 cells. To prioritize the miRNAs correlated with changes in autophagy, the miRNAs showing the same altered pattern under 5-FU treatment and starvation (a second standard means of autophagy induction) were considered more likely to be involved in the regulation of autophagy. The miRNAs showing the same altered pattern under these two conditions were 94 upregulated miRNAs and 22 downregulated miRNAs (S1 Table). The prediction of miRNA-regulated gene targets is a necessary step to understand the functions of a given miRNA. The intersection of two different programs (algorithms) was reported increasing the sensitivity of prediction. TargetScan identifies targets with conserved complementarity to the seed (nucleotides 2–7) of the miRNA [23]. We used the intersection of TargetScan and PicTar to predict the target genes of the altered miRNAs. If there was no data in PicTar, miRDB was used in place of PicTar. Overall, we identified and selected four downregulated miRNAs, hsa-miR-302a-3p, hsa-miR-548ah-5p, hsa-miR-133b and hsa-miR-323a-3p, and 27 upregulated miRNAs, hsa-miR-203a, hsa-miR-99b-5p, hsa-miR-195-5p, hsa-let-7c-5p, hsa-miR-320d, hsa-miR-301a-3p, vmiR-30e-5p, hsa-miR-374c-5p, hsa-miR-181a-5p, hsa-let-7g-5p, hsa-miR-513b-5p, hsa-miR-30b-5p, hsa-miR-19b-3p, hsa-miR-19a-3p, hsa-miR-15a-5p, hsa-miR-106b-5p, hsa-miR-330-3p, hsa-miR-582-5p, hsa-miR-16-5p, hsa-miR-30a-5p, hsa-miR-23a-3p, hsa-miR-26b-5p, hsa-miR-98-5p, hsa-miR-186-5p, hsa-miR-30d-5p, hsa-miR-93-5p and hsa-miR-320c, as having the predicted target genes involved in the regulation of autophagy, which include autophagy core genes and autophagy regulators (Table 2).

**Table 2:** Differential miRNA expression in starvation (Starv) vs. control (Ctrl) and 5-FU vs. control (DMSO) in HT29.

Human miRNA	Genomic location	Mean intensities in				Fold Change		Up/Down	(Predicted) target genes
		Ctrl	Starv	DMSO	5-FU	Starv vs Ctrl	5-FU vs DMSO		
miR-302a-3p	4q25	980	236	781	200	0.193	0.193	Down	ULK1
miR-548ah-5p	4	232	126	178	178	0.482	0.324	Down	ATG16L1, PRKAA2
miR-133b	6p12.2	182	62.5	144	97	0.163	0.449	Down	ATG14, GABARAPL1
miR-323a-3p	14q32.31	123	74	330	92.5	0.474	0.157	Down	ULK2
miR-203a	14q32.33	143.5	495	102	503.5	5.325	6.492	Up	ATG14
miR-99b-5p	19q13.41	212.5	1016.5	245	1311	6.243	5.624	Up	mTOR
miR-195-5p	17p13.1	72	193	40.5	80	7.235	4.151	Up	ATG14, PRKAR2A, BCL2
let-7c-5p	21q21.1	270.5	1144	96	767	5.127	10.67	Up	ULK2, TSC1, BCL2L1
miR-320d	13q14.11	93	214	32	167	4.208	118.3	Up	ATG14, ULK1
miR-301a-3p	17q22	332.5	1437.5	211	866.5	5.064	4.327	Up	ATG14, ATG16L1, ATG2B
miR-30e-5p	1p34.2	653	1239	361	1141	2.010	3.140	Up	BECN1, PIK3R2
miR-374c-5p	X	217.5	442.5	147	389	2.485	2.878	Up	ATG12, PIK3R1
miR-181a-5p	9q33.3	1237	2815	511	2055	2.367	3.935	Up	ATG10, PIK3R3, BCL2, MCL1
let-7g-5p	3p21.1	869	1966	584	1252	2.378	2.041	Up	ULK2, TSC1
miR-513b-5p	Xq27.3	93	178.5	89	188	3.424	2.407	Up	PIK3R3, BCL2L1
miR-30b-5p	8q24.22	798.5	1629.5	554	1519.5	2.150	2.652	Up	BECN1, PIK3R2
miR-19b-3p	Xq26.2	2596	5321.5	1400.5	3251	2.094	2.193	Up	ATG16L1, GABARAPL1
miR-19a-3p	13q31.3	2763	6281	1482.5	3761.5	2.324	2.399	Up	ATG16L1, PRKAA1,GABARAPL1
miR-15a-5p	13q14.2	885	2760	690.5	2115	3.290	2.941	Up	ATG14, PRKAR2A, BCL2
miR-106b-5p	7q22.1	1719	3814.5	1467	4103	2.285	2.648	Up	ATG16L1, ULK1, p62, MCL1
miR-330-3p	19q13.32	751	116.5	47	84	3.522	2.588	Up	PIK3R1
miR-582-5p	5q12.1	80	118.5	52	105.5	2.988	3.207	Up	ATG7, MCL1, RICTOR
miR-16-5p	3q25.33	996	5907.5	1283	3887.5	6.295	2.877	Up	ULK1, GABARAPL1, IFNG
miR-30a-5p	6q13	544.5	1115.5	138	978.5	2.196	8.175	Up	ATG5, BECN1, PRKAA1, GABARAPL2, PIK3R2
miR-23a-3p	19p13.13	1625.5	5023	936.5	5548	3.200	5.698	Up	ATG12. RPTOR, BCL2
miR-26b-5p	2q35	589	1554	325.5	1371	2.870	4.267	Up	ULK1, GABARAP, PIK3R3
miR-98-5p	Xp11.22	1011.5	2093.5	305.5	1252.5	2.184	4.227	Up	ULK2, PRKAA2, BCL2L1
miR-186-5p	1p31.1	493	1278.5	301.5	2110.5	2.828	7.175	Up	MCL1
miR-30d-5p	8q24.22	947.5	3032.5	590	2747.5	3.371	4.537	Up	ATG5, BECN1, PIK3R2
miR-93-5p	7q22.1	1234	4366	855.5	5398.5	3.696	6.084	Up	ATG16L1, ULK1, MCL1, p62
miR-320c	18q11.2	273	451.5	128.5	851	2.059	7.905	Up	ULK1, MAP1LC3B, ATG7

### Validation of microarray data using qRT-PCR in HT29, HCT11, and DLD1 cells

**Figure 3:**
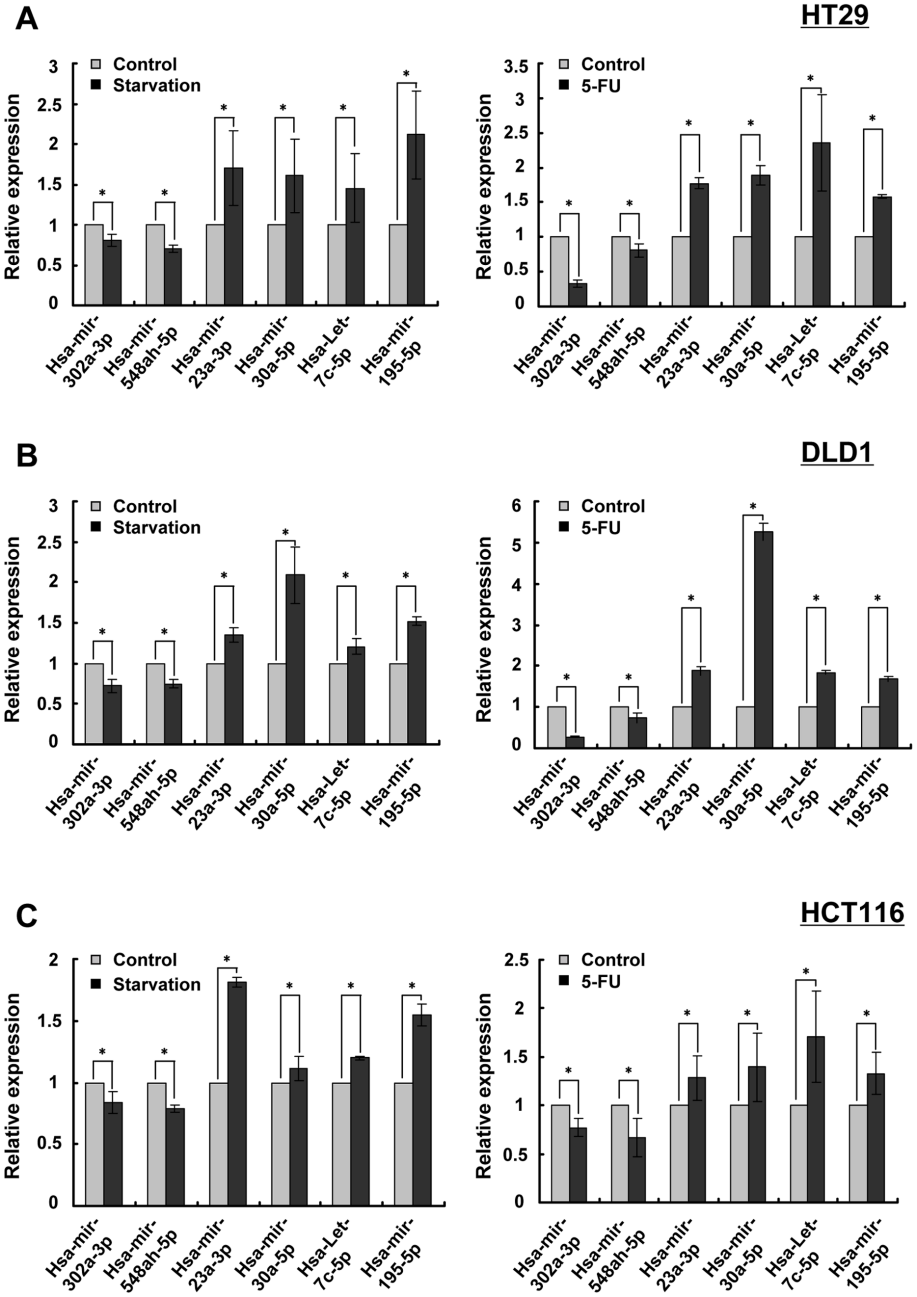
qRT-PCR validation of altered expression of miRNAs under 5-FU treatment and starvation in human colon cancer cells. Three kinds of human colon cancer cell lines, HT29 (A), DLD1 (B) and HCT116 (C), were treated as described in Fig. 2. qRT-PCR was performed to validate the alteration of the expression of hsa-miR-302a-3p, hsa-miR-548ah-5p, hsa-miR-30a-5p, hsa-miR-23-3p, hsa-miR-195a-5p and hsa-let-7c-5p under 5-FU treatment (5-FU) and starvation. Data are shown as the mean ±SD. * p<0.05. Experiments were repeated three times with reproducible results.

To validate the microarray data, we performed qRT-PCR on two downregulated (hsa-miR-302a-3p and has-miR-548ah-5p) and four upregulated miRNAs (hsa-miR-30a-5p, hsa-miR-23a-3p, hsa-miR-195-5p and hsa-let-7c-5p) in the 5-FU treated or starved HT29 cells. Because colon cancer is heterogeneous, the altered expression of these miRNAs was also determined in other two human colon cancer-derived cell lines, HCT116 and DLD1. We found that in accord with the results from miRNA microarray analysis the expression of these miRNAs changed significantly based on their qRT-PCR readings (Fig. 3).

### Pathway analysis and GO network analysis revealed the miRNAs-autophagy interconnection

**Figure 4:**
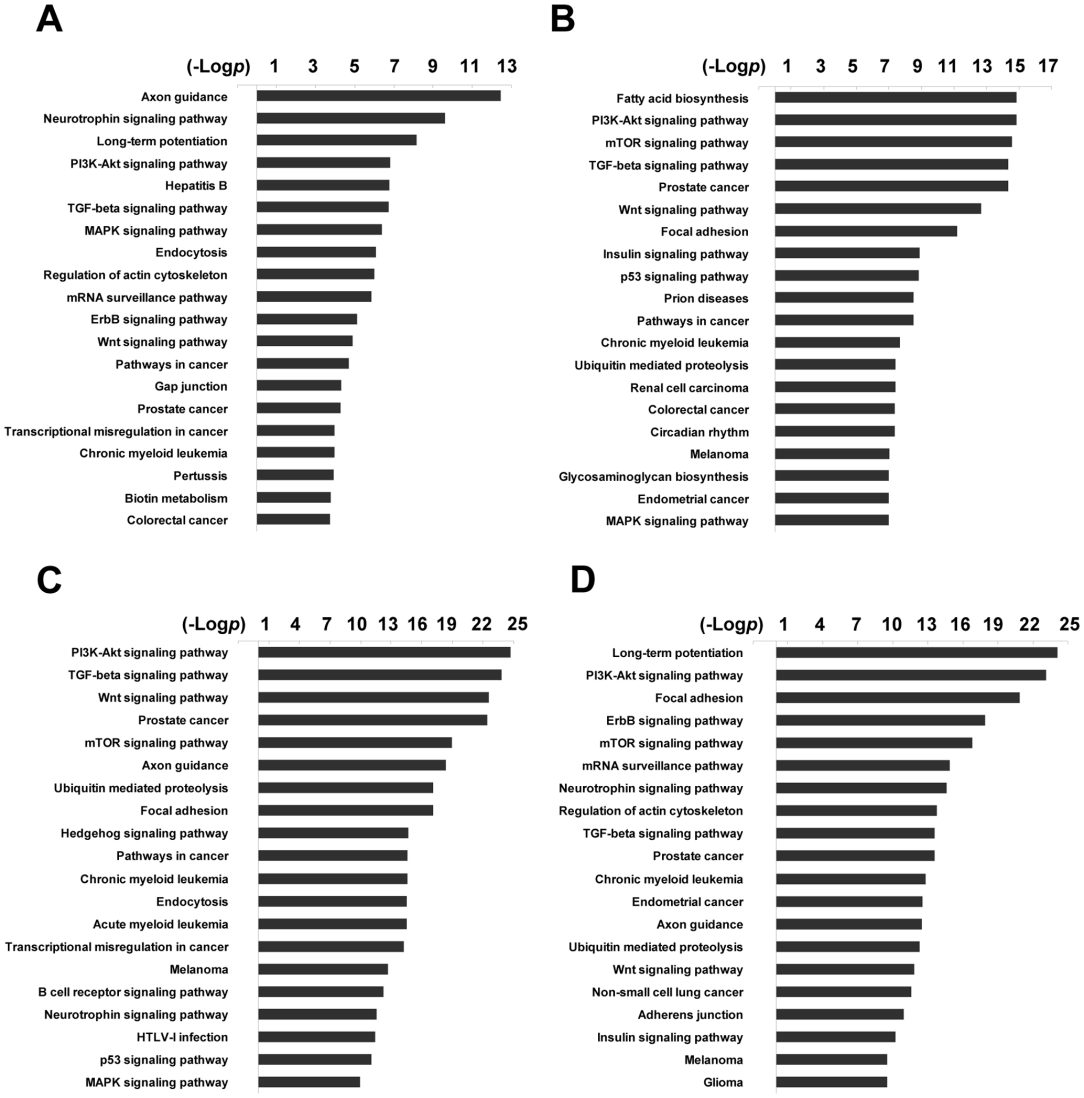
Bioinformatics pathway analysis based on the target genes of the selected miRNAs. DIANA-miRPath v2.0 was applied to analyze the main functions of the selected 31 miRNAs (A, the four downregulated miRNAs; B, miRNAs upregulated more than four-fold under 5-FU treatment and starvation; C, miRNAs upregulated between two- and four-fold under two conditions; D, miRNAs upregulated more than four-fold and between two- and four-fold). The vertical axis is the KEGG pathway category, and the horizontal axis is the negative logarithm of the *p* value (-Log *p*), which represents the significance of the pathways.

**Figure 5:**
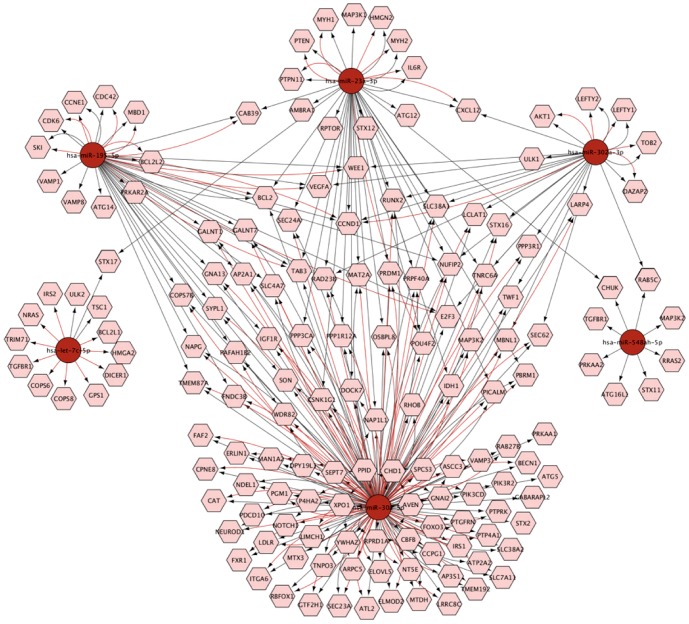
Graph depicting the network of mRNA targets likely to be regulated by six miRNAs. The integrative networks were created using Cytoscape software. Each network includes two types of nodes, individual miRNAs (red circles) and their predicted mRNA targets (pink hexagon), obtained from two different public databases (miRTarBase and Targetscan). The graph shows that the mRNA targets appear in two databases, which are identified by the color of the connecting arrows, miRTarBase (red) and Targetscan (black).

To gain insight into the functions of these miRNAs, DIANA-miRPath was used to analyze KEGG pathways influenced by these 31 miRNAs (Fig. 4). As a result, the high significant enrichment pathways of the four downregulated miRNAs included the MAPK signaling pathway, which is reported to positively participate in the regulation of autophagy [24] (Fig. 4A). More interestingly, among the high significant enrichment pathways of the 27 upregulated miRNAs, the mTOR signaling pathway was significantly identified by these miRNAs (Fig. 4B, 4C and 4D). Consistently, the protein level of mTOR was decreased under these two conditions (Fig. 2C). Additionally, miRNA-mRNA gene network analysis integrated these miRNAs and GOs by outlining the interactions of miRNA and GO-related genes using Cytoscape software (Fig. 5).

## Discussion

5-FU-based chemotherapy is the mainstream of the adjuvant treatment of CRC. Autophagy modulation has been considered as a potential strategy to implement chemotherapy in tumor therapy [4]. MiRNAs play important roles in controlling cellular functions and have been reported to be involved in the regulation of autophagy in recent years [25]. In our experiment, induction of autophagy was confirmed in HT29 cells by both 5-FU treatment and nutrient starvation. Using miRNA microarray analysis, qRT-PCR, and bioinformatics, we identified and selected four downregulated miRNAs including hsa-miR-302a-3p and 27 upregulated miRNAs including hsa-miR-30a-5p, hsa-miR-23a-3p, hsa-miR-195a-5p, hsa-miR-99b-5p and hsa-let-7c-5p under these two conditions as having the potential to target genes involved in the regulation of autophagy (Table 2). Further functional analyses of these miRNAs needs to be performed.

Accumulating evidence suggests that autophagy plays important roles in tumorigenesis and tumor therapy [3]. It can either inhibit or promote tumorigenesis depending on the stage of the tumor. As to tumor therapy, autophagy appears to mediate the effect of anti-cancer agents as the inhibition of autophagy suppresses their therapeutic effectiveness. Autophagy also can be activated as a pro-survival response to promote therapeutic resistance to cytotoxic therapy. And the inhibition of autophagy enhances drug- or radiation-induced cell death as we have reported [6], [7]. Molecules involved in the regulation of the autophagic process have emerged as promising targets for innovative anticancer therapies [4].

Autophagy (mainly macroautophagy) is a tightly regulated, conserved catabolic process. After induction, parts of the cytoplasm are sequestered into characteristic double-membrane vesicles known as autophagosomes (vesicle nucleation, vesicle elongation and retrieval). Subsequently, autophagosomes fuse with late endosomes or lysosomes, forming the autolysosome (fusion). Exposure of the inner compartment to lysosomal hydrolases causes degradation of the cytoplasmic cargo, and the resulting degradation products are then released into the cytosol for recycling. Tight control of autophagy is essential for cell homeostasis and response to cellular stress. A large family of core autophagy regulators, the AuTophaGy (ATG)-related genes, serves to coordinately regulate the stepwise progression of autophagy from autophagy induction to vesicle nucleation, vesicle elongation, retrieval and fusion [26]. In addition, a diverse and complex network of upstream signaling pathways contribute to autophagy regulation including the phosphatidylinositol 3 kinase (PI3K), RAS-proto-oncogene and AMP-activated protein kinase (AMPK) pathways, many of which converge at the mammalian target of rapamycin complex 1 (mTORC1), a key negative regulator of autophagy signaling [27].

In our experiment, 27 miRNAs that potentially target genes regulating autophagy were found to be upregulated after 5-FU treatment or starvation. Pathway analysis suggested that the mTOR signaling pathway was significantly identified by these miRNAs. It was previously demonstrated in breast cancer cells that nutrient starvation results in an increase in autophagy through inhibition of mTOR [28]. Our results also strongly supported this effect during 5-FU-induced autophagy in colon cancer cells. Among these miRNAs, the predicted target genes of hsa-miR-99b-5p included mTOR. And the increase of this miRNA upon two types of autophagy induction (5-FU treatment and starvation) was significant, 5.624 and 6.243 times higher than the control. Hsa-miR-99b-5p warrants further investigation in the regulation of autophagy in 5-FU treatment in human colon cancer. In addition to the mTOR network, the beclin1 network was also reported to regulate autophagy in breast cancer [29]. The Bcl2 family blocks starvation-induced autophagy by interacting with the BH3 domain of Beclin1 and are negative regulators of autophagy. In our experiment, hsa-let-7c-5p, hsa-miR-195-5p, hsa-miR-23a-3p, hsa-miR-15a-5p, hsa-miR-98-5p, and hsa-miR-181a-5p are predicted to target genes in the Bcl2 family and also warrant further investigation. Although these 27 miRNAs showed upregulated expression under these two methods of autophagy inductions, they are predicted to target autophagy core genes; hsa-miR-30a-5p targeting of BECN1 and ATG5 has been demonstrated in the previous reports [30]. The function of these miRNAs needs to be further investigated. In addition, there were only four downregulated miRNAs with predicted targets involved in autophagy regulation, less than the quantity of upregulated miRNAs. It also demonstrated the importance of the mTOR signaling pathway in the regulation of autophagy. Moreover, predicted target genes included autophagy core genes; hsa-miR-302a-3p targets ULK1 and hsa-miR-548ah-5p targets ATG16L1, suggesting that these four miRNAs participate in the autophagy process in 5-FU treatment and have the potential to be used to manipulate autophagy in 5-FU based chemotherapy in CRC.

The roles of autophagy in cancer are dependent on the type of cancer, the context and the location [4]. In this study, we focused on 5-FU-induced autophagy in human colon cancer based on our and other previous reports. There had 4 down-regulated miRNAs including hsa-miR-302a-3p and hsa-miR-548ah-5p; and 27 up-regulated miRNAs including hsa-let-7c-5p and hsa-miR-30a-5p upon 5-FU treatment and starvation in human colon cancer cells. These 31 miRNAs have the predicted target genes of the regulation of autophagy, including autophagy core genes and autophagy regulators and are promising targets for autophagy modulation in 5-FU-based chemotherapy in CRC. These data could also shed light on miRNAs-autophagy interactions in other cancer types as well as provide a mechanism to potentially regulate autophagy in clinical practice.

## Supporting Information

S1 Fig **5-FU induces little apoptosis in HT29 cells.** HT29 cells were incubated with 5-FU for 24 h. Flow cytometry using Annexin V and PI was performed to detect apoptosis in our experiment. There was little apoptosis of HT29 cells after 5-FU treatment for 24 h.
(TIF)

S1 Table Differential miRNA expression in starvation (Starv) vs. control (Ctrl) and 5-FU vs. control (DMSO) in HT29.
(DOC)

## References

[1] O'Connell MJ (2009) Oxaliplatin or irinotecan as adjuvant therapy for colon cancer: the results are in. J Clin Oncol 27:3082–4. 19451420 10.1200/JCO.2009.22.2919

[2] Eskelinen EL, Saftig P (2009) Autophagy: A lysosomal degradation pathway with a central role in health and disease. BBA 1793:664–673. 18706940 10.1016/j.bbamcr.2008.07.014

[3] Lorin S, Hamaï A, Mehrpour M, Codogno P (2013) Autophagy regulation and its role in cancer. Semin Cancer Biol 23:361–79. 23811268 10.1016/j.semcancer.2013.06.007

[4] Meschini S, Condello1 M, Lista P, Arancia G (2011) Autophagy: Molecular Mechanisms and their Implications for Anticancer therapies. Curr Cancer Drug Tar 11:357–79. 10.2174/156800911794519707 21247381

[5] Chen S, Rehman SK, Zhang W, Wen A, Yao L, et al (2010) Autophagy is a therapeutic target in anticancer drug resistance. BBA 1806:220–9. 20637264 10.1016/j.bbcan.2010.07.003

[6] Li J, Hou N, Faried A, Tsutsumi S, Takeuchi T, et al (2009) Inhibition of autophagy by 3-MA enhances the effect of 5-FU-induced apoptosis in colon cancer cells. Ann Surg Oncol 16:761–71. 19116755 10.1245/s10434-008-0260-0

[7] Li J, Hou N, Faried A, Tsutsumi S, Kuwano H (2010) Inhibition of autophagy augments 5-fluorouracil chemotherapy in human colon cancer in vitro and in vivo model. Eur J Cancer 46:1900–9. 20231086 10.1016/j.ejca.2010.02.021

[8] He L, Hannon GJ (2004) MicroRNA: small RNAs with a big role in gene regulation. Nat Rev Genet 5:522–31. 15211354 10.1038/nrg1379

[9] Marsit CJ, Eddy K, Kelsey KT (2006) microRNA responses to cellular stress. Cancer Res 66:1084–8. 10.1158/0008-5472.CAN-06-1894 17108120

[10] Babar IA, Slack FJ, Weidhaas JB (2008) miRNA modulation of the cellular stress response. Future Oncol 4:289–98. 18407740 10.2217/14796694.4.2.289

[11] Kulshrehtha R, Ferracin M, Wojcik SE, Garzon R, Alder H, et al (2007) A microRNA signature of hypoxia. Mol Cell Biol 27:1859–67. 17194750 10.1128/MCB.01395-06 PMC1820461

[12] Zou Z, Wu L, Ding H, Wang Y, Zhang Y, et al (2012) MicroRNA-30a Sensitizes Tumor Cells to cis-platinum via suppressing beclin 1-mediated Autophagy. J Biol Chem 287:4148–56. 22157765 10.1074/jbc.M111.307405 PMC3281695

[13] Frankel LB, Wen J, Lees M, Høyer-Hansen M, Farkas T, et al (2011) MicroRNA-101 is a potent inhibitor of autophagy. EMBO J 30:4628–41. 21915098 10.1038/emboj.2011.331 PMC3243595

[14] Jegga AG, Schneider L, Ouyang X, Zhang J (2011) Systems biology of the autophagy- lysosomal pathway. Autophagy 7:477–89. 21293178 10.4161/auto.7.5.14811 PMC3127210

[15] Yin JQ, Zhao RC, Morris KV (2008) Profiling microRNA expression with microarrays. Trends Biotechnol 26:70–6. 18191262 10.1016/j.tibtech.2007.11.007

[16] Kubota C, Torii S, Hou N, Saito N, Yoshimoto Y, et al (2010) Constitutive reactive oxygen species generation from autophagosome/lysosome in neuronal oxidative toxicity. J Biol Chem 285:667–94. 19850931 10.1074/jbc.M109.053058 PMC2804214

[17] Lewis BP, Shih I, Jones-Rhoades MW, Bartel DP, Burge CB (2003) Prediction of Mammalian MicroRNA Targets. Cell 115:787–98. 14697198 10.1016/s0092-8674(03)01018-3

[18] Krek A, Grün D, Poy MN, Wolf R, Rosenberg L, et al (2005) Combinatorial microRNA target predictions. Nat Genet 37:495–500. 15806104 10.1038/ng1536

[19] Wang X, El Naqa IM (2008) Prediction of both conserved and nonconserved microRNA targets in animals. Bioinformatics 24(3):325–332. 18048393 10.1093/bioinformatics/btm595

[20] Vlachos IS, Kostoulas N, Vergoulis T, Georgakilas G, Reczko M, et al (2012) DIANA miRPath v.2.0: investigating the combinatorial effect of microRNAs in pathways. Nucleic Acids Res 40:W498–504. 22649059 10.1093/nar/gks494 PMC3394305

[21] Kutmon M, Kelder T, Mandaviya P, Evelo CT, Coort SL (2013) CyTargetLinker: a cytoscape app to integrate regulatory interactions in network analysis. PLoS One 8:e82160.. 24340000 10.1371/journal.pone.0082160 PMC3855388

[22] Pérez-Rivas LG, Jerez JM, Carmona R, de Luque V, Vicioso L, et al (2014) A microRNA signature associated with early recurrence in breast cancer. PLoS One 9:e91884.. 24632820 10.1371/journal.pone.0091884 PMC3954835

[23] Sethupathy P, Megraw M, Hatzigeorgiou AG (2006) A guide through present computational approaches for the identification of mammalian microRNA targets. Nat methods 3:881–6. 17060911 10.1038/nmeth954

[24] Chen KL, Chang WS, Cheung CH, Lin CC, Huang CC, et al (2012) Targeting cathepsin S induces tumor cell autophagy via the EGFR-ERK signaling pathway. Cancer Lett 317:89–98. 22101325 10.1016/j.canlet.2011.11.015

[25] Zhai H, Fesler A, Ju J (2013) MicroRNA: a third dimension in autophagy. Cell Cycle 12:246–50. 23255136 10.4161/cc.23273 PMC3575453

[26] Mizushima N, Yoshimori T, Ohsumi Y (2011) The role of Atg proteins in autophagosome formation. Annu Rev Cell Dev Biol 27:107–32. 21801009 10.1146/annurev-cellbio-092910-154005

[27] He C, Klionsky DJ (2009) Regulation Mechanisms and Signaling Pathways of Autophagy. Annu Rev Genet 43:67–93. 19653858 10.1146/annurev-genet-102808-114910 PMC2831538

[28] Levine B, Kroemer G (2008) Autophagy in the pathogenesis of disease. Cell 132:27–42. 18191218 10.1016/j.cell.2007.12.018 PMC2696814

[29] Pattingre S, Tassa A, Qu X, Garuti R, Liang XH, et al (2005) Bcl-2 antiapoptotic proteins inhibit beclin 1-dependent autophagy. Cell 122:927–39. 16179260 10.1016/j.cell.2005.07.002

[30] Yu Y, Yang L, Zhao M, Zhu S, Kang R, et al (2012) Targeting microRNA-30a-mediated autophagy enhances imatinib activity against human chronic myeloid leukemia cells. Leukemia 26:1752–60. 22395361 10.1038/leu.2012.65

