# Supplementary figures and images for: MicroRNA Profiling in Human Colon Cancer Cells during 5-Fluorouracil-Induced Autophagy
Source: PLoS One. 2014 Dec 19;9(12):e114779. doi: 10.1371/journal.pone.0114779 (PMC4272278; doi:10.1371/journal.pone.0114779)

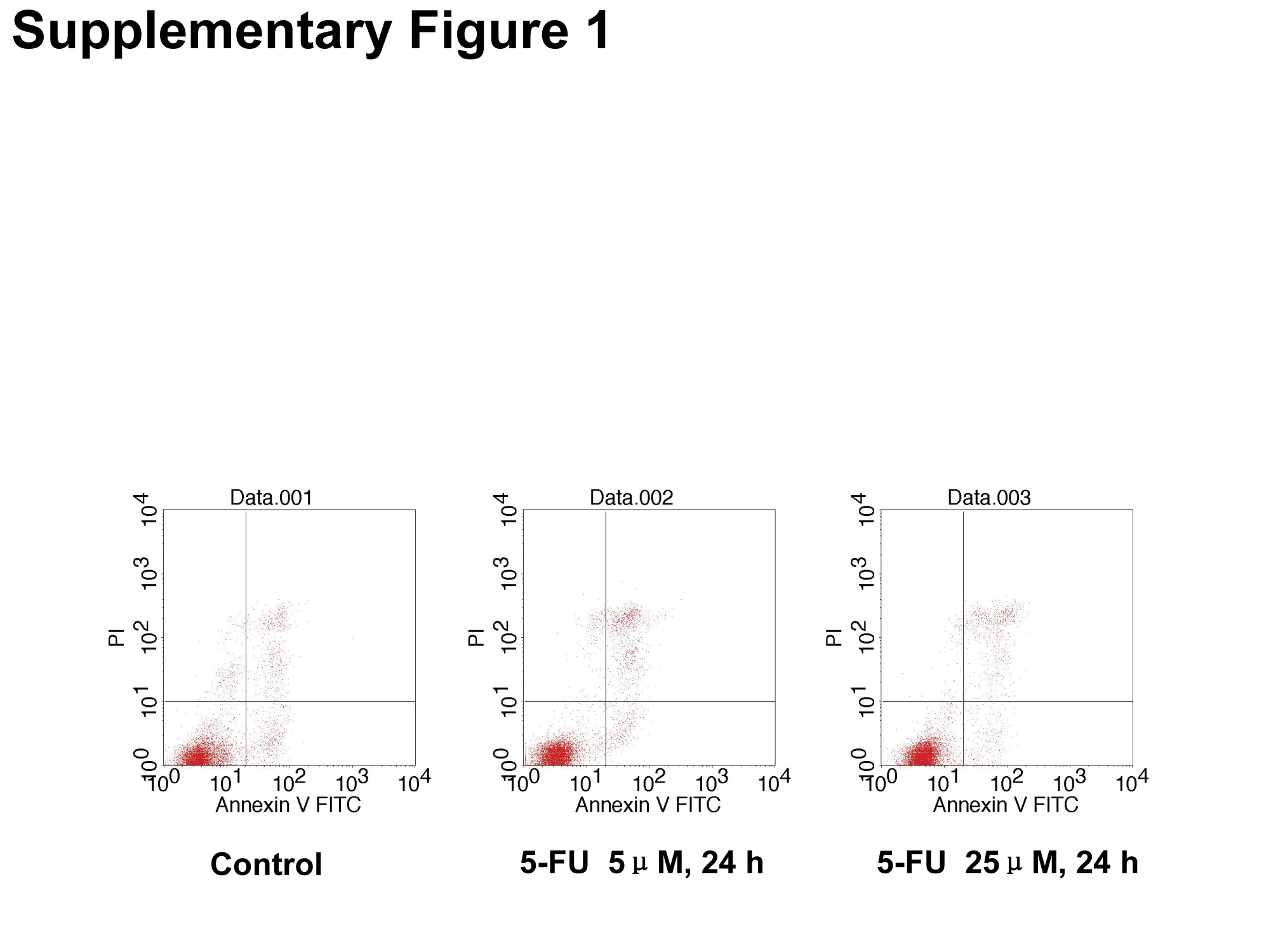

Supplement: S1 Fig — 5-FU induces little apoptosis in HT29 cells. HT29 cells were incubated with 5-FU for 24 h. Flow cytometry using Annexin V and PI was performed to detect apoptosis in our experiment. There was little apoptosis of HT29 cells after 5-FU treatment for 24 h. (TIF) [file pone.0114779.s001.tif]
