# Supplementary material for: MicroRNA Profiling in Human Colon Cancer Cells during 5-Fluorouracil-Induced Autophagy
Source: PLoS One. 2014 Dec 19;9(12):e114779. doi: 10.1371/journal.pone.0114779 (PMC4272278; doi:10.1371/journal.pone.0114779)
Supplement: S1 Table — Differential miRNA expression in starvation (Starv) vs. control (Ctrl) and 5-FU vs. control (DMSO) in HT29. (DOC) [file pone.0114779.s002.doc]

| **Human miRNA** | **Mean intensities in** | | | |  | **Fold Change** | | **Up/Down** |
| --- | --- | --- | --- | --- | --- | --- | --- | --- |
|  | **Ctrl** | **Starv** | **DMSO** | **5-FU** |  | **Starv vs Ctrl** | **5-FU vs DMSO** |  |
| **miR-302a-3p** | 979.5 | 236 | 780.5 | 200 |  | **0.193** | **0.193** | **Down** |
| **miR-639** | 959 | 130 | 1259 | 133 |  | **0.09** | **0.07** | **Down** |
| **miR-219a-1-3p** | 104 | 49 | 353 | 77.5 |  | **0.12** | **0.1** | **Down** |
| **miR-377-5p** | 322 | 74 | 693 | 117 |  | **0.13** | **0.11** | **Down** |
| **miR-411-3p** | 100 | 52.5 | 778 | 91 |  | **0.22** | **0.06** | **Down** |
| **miR-630** | 793 | 93 | 1186 | 141 |  | **0.07** | **0.08** | **Down** |
| **miR-374b-3p** | 333 | 95.5 | 764 | 133 |  | **0.19** | **0.12** | **Down** |
| **miR-485-3p** | 490 | 96.5 | 670 | 140 |  | **0.12** | **0.14** | **Down** |
| **miR-147b** | 350 | 67.5 | 982 | 119 |  | **0.09** | **0.07** | **Down** |
| **miR-30e-3p** | 290 | 155 | 587 | 208 |  | **0.49** | **0.28** | **Down** |
| **miR-548ah-5p** | 231.5 | 126 | 177.5 | 177.5 |  | **0.482** | **0.324** | **Down** |
| **miR-133b** | 182 | 62.5 | 143.5 | 97 |  | **0.163** | **0.449** | **Down** |
| **miR-494-5p** | 96.5 | 56 | 302 | 102 |  | **0.32** | **0.19** | **Down** |
| **miR-548at-5p** | 120 | 61 | 383 | 68.5 |  | **0.28** | **0.07** | **Down** |
| **miR-212-3p** | 103 | 64 | 492 | 93.5 |  | **0.4** | **0.1** | **Down** |
| **miR-372-5p** | 133 | 68 | 1061 | 151 |  | **0.36** | **0.1** | **Down** |
| **miR-495-5p** | 399 | 152 | 1379 | 201 |  | **0.32** | **0.11** | **Down** |
| **miR-628-3p** | 258 | 135 | 525 | 133 |  | **0.46** | **0.17** | **Down** |
| **miR-323a-3p** | 123 | 74 | 330 | 92.5 |  | **0.474** | **0.157** | **Down** |
| **miR-221-5p** | 190 | 103 | 1003 | 87 |  | **0.44** | **0.04** | **Down** |
| **miR-550b-3p** | 312 | 142 | 987 | 169 |  | **0.38** | **0.12** | **Down** |
| **miR-758-5p** | 113 | 58.5 | 265 | 104 |  | **0.31** | **0.23** | **Down** |
| **miR-550b-2-5p** | 98 | 433 | 46 | 287 |  | **8.82** | **16.2** | **Up** |
| **miR-378g** | 58 | 113 | 37 | 134 |  | **12.8** | **16.8** | **Up** |
| **miR-203a** | 144 | 495 | 102 | 503.5 |  | **5.33** | **6.49** | **Up** |
| **miR-577** | 66 | 77.5 | 41.5 | 90.5 |  | **7.18** | **6.55** | **Up** |
| **miR-221-3p** | 1267 | 5693 | 842 | 6124 |  | **4.73** | **7.05** | **Up** |
| **miR-99b-5p** | 213 | 1017 | 245 | 1311 |  | **6.24** | **5.62** | **Up** |
| **miR-182-3p** | 61 | 88.5 | 39 | 117 |  | **7.07** | **14.9** | **Up** |
| **miR-222-3p** | 1029 | 9543 | 548 | 12904 |  | **9.86** | **23.4** | **Up** |
| **miR-195-5p** | 72 | 193 | 40.5 | 80 |  | **7.24** | **4.15** | **Up** |
| **let-7c-5p** | 271 | 1144 | 96 | 767 |  | **5.13** | **10.7** | **Up** |
| **miR-7-5p** | 1530 | 7107 | 1497 | 8293 |  | **4.84** | **5.28** | **Up** |
| **miR-98-3p** | 77 | 158 | 52.5 | 170 |  | **4.98** | **7.14** | **Up** |
| **miR-320d** | 93 | 214 | 32 | 167 |  | **4.21** | **118** | **Up** |
| **miR-301a-3p** | 333 | 1438 | 211 | 866.5 |  | **5.06** | **4.33** | **Up** |
| **let-7a-3p** | 98.5 | 269 | 62.5 | 195 |  | **5.08** | **4.97** | **Up** |
| **miR-146a-5p** | 66.5 | 116 | 41 | 154 |  | **5.23** | **17.1** | **Up** |
| **miR-194-3p** | 61 | 75.5 | 38 | 95 |  | **4.24** | **10.9** | **Up** |
| **miR-188-5p** | 59.5 | 77 | 37.5 | 82 |  | **5.55** | **11.7** | **Up** |
| **miR-27b-3p** | 542 | 1490 | 407 | 1005 |  | **2.98** | **2.39** | **Up** |

**TableS1**  **Differential miRNA expression in starvation (Starv) vs. control (Ctrl) and 5-FU vs. control (DMSO) in HT29**

| **Human miRNA** | **Mean intensities in** | | | |  | **Fold Change** | | **Up/Down** |
| --- | --- | --- | --- | --- | --- | --- | --- | --- |
|  | **Ctrl** | **Starv** | **DMSO** | **5-FU** |  | **Starv vs Ctrl** | **5-FU vs DMSO** |  |
| **miR-30e-5p** | 653 | 1239 | 361 | 1141 |  | **2.01** | **3.14** | **Up** |
| **miR-92b-3p** | 247 | 459 | 115 | 317 |  | **2.13** | **3.2** | **Up** |
| **miR-374c-5p** | 218 | 443 | 147 | 389 |  | **2.48** | **2.88** | **Up** |
| **miR-181a-5p** | 1237 | 2815 | 511 | 2055 |  | **2.37** | **3.93** | **Up** |
| **let-7g-5p** | 869 | 1966 | 584 | 1252 |  | **2.38** | **2.04** | **Up** |
| **miR-744-5p** | 179 | 481 | 109 | 256 |  | **3.51** | **2.54** | **Up** |
| **miR-196a-5p** | 397 | 748 | 262 | 553 |  | **2.08** | **2.06** | **Up** |
| **miR-29b-3p** | 3867 | 9010 | 2147 | 6472 |  | **2.37** | **2.84** | **Up** |
| **miR-941** | 97.5 | 163 | 70 | 166 |  | **2.84** | **2.98** | **Up** |
| **miR-513b-5p** | 93 | 179 | 89 | 188 |  | **3.42** | **2.41** | **Up** |
| **miR-182-5p** | 174 | 521 | 143 | 341 |  | **3.98** | **2.51** | **Up** |
| **miR-194-5p** | 1213 | 3174 | 1178 | 2696 |  | **2.73** | **2.17** | **Up** |
| **miR-335-5p** | 218 | 457 | 166 | 504 |  | **2.63** | **3.21** | **Up** |
| **miR-107** | 673 | 1772 | 319 | 840 |  | **2.81** | **2.59** | **Up** |
| **miR-19b-3p** | 2596 | 5322 | 1401 | 3251 |  | **2.09** | **2.19** | **Up** |
| **miR-19a-3p** | 2763 | 6281 | 1483 | 3762 |  | **2.32** | **2.4** | **Up** |
| **miR-24-2-5p** | 79.5 | 126 | 55 | 142 |  | **3.27** | **3.95** | **Up** |
| **miR-125a-3p** | 101 | 148 | 93.5 | 229 |  | **2.11** | **2.8** | **Up** |
| **miR-15a-5p** | 885 | 2760 | 691 | 2115 |  | **3.29** | **2.94** | **Up** |
| **miR-106b-5p** | 1719 | 3815 | 1467 | 4103 |  | **2.28** | **2.65** | **Up** |
| **miR-18a-3p** | 87.5 | 159 | 71 | 168 |  | **3.4** | **3.06** | **Up** |
| **miR-130b-5p** | 78.5 | 105 | 57 | 148 |  | **2.8** | **3.95** | **Up** |
| **miR-330-3p** | 75 | 117 | 47 | 84 |  | **3.52** | **2.59** | **Up** |
| **miR-338-3p** | 534 | 1313 | 267 | 947 |  | **2.68** | **3.65** | **Up** |
| **miR-582-5p** | 80 | 119 | 52 | 106 |  | **2.99** | **3.21** | **Up** |
| **miR-30b-5p** | 799 | 1630 | 554 | 1520 |  | **2.15** | **2.65** | **Up** |
| **miR-27a-3p** | 1117 | 5027 | 719 | 2517 |  | **4.75** | **3.36** | **Up** |
| **miR-208b-3p** | 82 | 273 | 84 | 161 |  | **8.15** | **2.11** | **Up** |
| **miR-452-5p** | 168 | 524 | 201 | 744 |  | **4.27** | **3.94** | **Up** |
| **miR-16-5p** | 996 | 5908 | 1283 | 3888 |  | **6.29** | **2.88** | **Up** |
| **miR-149-5p** | 88 | 132.5 | 47 | 149.5 |  | **2.54** | **6.3** | **Up** |
| **miR-30a-5p** | 545 | 1115.5 | 138 | 978.5 |  | **2.2** | **8.17** | **Up** |
| **miR-302c-5p** | 172 | 312 | 61.5 | 342.5 |  | **2.45** | **10.7** | **Up** |
| **miR-151a-3p** | 522 | 1648 | 282 | 2686 |  | **3.52** | **9.98** | **Up** |
| **miR-23a-3p** | 1626 | 5023 | 937 | 5548 |  | **3.2** | **5.7** | **Up** |
| **miR-26b-5p** | 589 | 1554 | 326 | 1371 |  | **2.87** | **4.27** | **Up** |
| **miR-26a-2-3p** | 77.5 | 83 | 44 | 111.5 |  | **2.02** | **5.16** | **Up** |
| **miR-542-3p** | 98 | 174 | 43 | 143.5 |  | **3.1** | **7.87** | **Up** |
| **miR-550a-5p** | 106 | 241.5 | 46.5 | 184 |  | **3.84** | **9.1** | **Up** |
| **miR-30b-3p** | 65.5 | 75 | 40 | 100.5 |  | **2.69** | **6.61** | **Up** |

**TableS1 Continued**

**Table S1 Continued**

| **Human miRNA** | **Mean intensities in** | | | |  | **Fold Change** | | **Up/Down** |
| --- | --- | --- | --- | --- | --- | --- | --- | --- |
|  | **Ctrl** | **Starv** | **DMSO** | **5-FU** |  | **Starv vs Ctrl** | **5-FU vs DMSO** |  |
| **miR-210-3p** | 206 | 532.5 | 176 | 792.5 |  | **3.24** | **4.89** | **Up** |
| **miR-17-3p** | 210 | 591.5 | 121 | 450.5 |  | **3.64** | **4.43** | **Up** |
| **miR-98-5p** | 1012 | 2093.5 | 306 | 1253 |  | **2.18** | **4.23** | **Up** |
| **miR-10b-5p** | 863 | 1644 | 88 | 660.5 |  | **2.04** | **10.4** | **Up** |
| **miR-200b-5p** | 125 | 294.5 | 88.5 | 327.5 |  | **3.73** | **4.74** | **Up** |
| **miR-378d** | 128 | 208 | 73.5 | 372.5 |  | **2.42** | **8.05** | **Up** |
| **miR-320c** | 273 | 451.5 | 129 | 851 |  | **2.06** | **7.91** | **Up** |
| **miR-550a-3-5p** | 426 | 1409.5 | 60.5 | 927 |  | **3.77** | **29.6** | **Up** |
| **miR-30d-5p** | 948 | 3032.5 | 590 | 2748 |  | **3.37** | **4.54** | **Up** |
| **miR-186-5p** | 493 | 1278.5 | 302 | 2111 |  | **2.83** | **7.17** | **Up** |
| **miR-30d-3p** | 73.5 | 103 | 39 | 84.5 |  | **2.58** | **6.04** | **Up** |
| **miR-641** | 63 | 75.5 | 38 | 128.5 |  | **2.5** | **12.1** | **Up** |
| **miR-556-3p** | 75 | 111.5 | 43.5 | 161.5 |  | **3.17** | **9.68** | **Up** |
| **miR-543** | 68 | 81.5 | 35.5 | 108.5 |  | **2.75** | **20.4** | **Up** |
| **miR-138-2-3p** | 1299 | 2585.5 | 426 | 2825 |  | **2.06** | **6.61** | **Up** |
| **miR-548am-5p** | 77.5 | 95.5 | 40 | 114.5 |  | **2.21** | **9.35** | **Up** |
| **miR-32-5p** | 261 | 739 | 92.5 | 539.5 |  | **3.52** | **8.04** | **Up** |
| **miR-339-3p** | 77 | 116 | 58.5 | 174.5 |  | **3.09** | **4.75** | **Up** |
| **miR-671-5p** | 105 | 141.5 | 65.5 | 248.5 |  | **2.03** | **5.93** | **Up** |
| **let-7e-3p** | 75.5 | 98.5 | 45 | 124 |  | **2.52** | **5.79** | **Up** |
| **miR-22-3p** | 1089 | 3332.5 | 744 | 3859 |  | **3.2** | **5.01** | **Up** |
| **miR-345-5p** | 76 | 103 | 48.5 | 206 |  | **2.91** | **9.84** | **Up** |
| **miR-331-3p** | 336 | 1117 | 303 | 1523 |  | **3.89** | **5.15** | **Up** |
| **miR-151a-5p** | 379 | 1105.5 | 290 | 1228 |  | **3.33** | **4.34** | **Up** |
| **miR-93-5p** | 1234 | 4366 | 856 | 5399 |  | **3.7** | **6.08** | **Up** |
| **miR-339-5p** | 274 | 728.5 | 173 | 1229 |  | **3.25** | **8.03** | **Up** |
| **miR-185-5p** | 211 | 629 | 144 | 1030 |  | **3.78** | **8.46** | **Up** |
| **miR-197-3p** | 186 | 533 | 96.5 | 426.5 |  | **3.79** | **5.76** | **Up** |
| **miR-93-3p** | 90 | 164 | 51 | 281.5 |  | **3.29** | **13.4** | **Up** |
| **miR-652-3p** | 153 | 411.5 | 108 | 544 |  | **3.89** | **6.45** | **Up** |
| **miR-598-3p** | 95.5 | 135 | 37.5 | 151 |  | **2.23** | **28.5** | **Up** |
| **miR-374b-5p** | 373 | 932 | 237 | 999 |  | **2.81** | **4.41** | **Up** |
| **miR-103a-2-5p** | 91.5 | 126 | 41.5 | 157.5 |  | **2.23** | **16.5** | **Up** |
| **miR-200a-3p** | 4491 | 13719 | 1573 | 18692 |  | **3.11** | **11.3** | **Up** |
| **miR-451b** | 608 | 1970 | 452 | 1883 |  | **3.51** | **4.12** | **Up** |
